# Developmental stage-specific distribution and phosphorylation of Mblk-1, a transcription factor involved in ecdysteroid-signaling in the honey bee brain

**DOI:** 10.1038/s41598-020-65327-z

**Published:** 2020-05-26

**Authors:** Hitomi Kumagai, Takekazu Kunieda, Korefumi Nakamura, Yasuhiro Matsumura, Manami Namiki, Hiroki Kohno, Takeo Kubo

**Affiliations:** Department of Biological Sciences, Graduate School of Science, The University of Tokyo, Bunkyo-ku, Tokyo, 113-0033 Japan

**Keywords:** Molecular neuroscience, Transcription

## Abstract

In the honey bee, the mushroom bodies (MBs), a higher-order center in insect brain, comprise interneurons termed Kenyon cells (KCs). We previously reported that *Mblk-1*, which encodes a transcription factor involved in ecdysteroid-signaling, is expressed preferentially in the large-type KCs (lKCs) in the pupal and adult worker brain and that phosphorylation by the Ras/MAPK pathway enhances the transcriptional activity of Mblk-1 *in vitro*. In the present study, we performed immunoblotting and immunofluorescence studies using affinity-purified anti-Mblk-1 and anti-phosphorylated Mblk-1 antibodies to analyze the distribution and phosphorylation of Mblk-1 in the brains of pupal and adult workers. Mblk-1 was preferentially expressed in the lKCs in both pupal and adult worker brains. In contrast, some Mblk-1 was phosphorylated almost exclusively in the pupal stages, and phosphorylated Mblk-1 was preferentially expressed in the MB neuroblasts and lKCs in pupal brains. Immunofluorescence studies revealed that both Mblk-1 and phosphorylated Mblk-1 are located in both the cytoplasm and nuclei of the lKC somata in the pupal and adult worker brains. These findings suggest that Mblk-1 plays a role in the lKCs in both pupal and adult stages and that phosphorylated Mblk-1 has pupal stage-specific functions in the MB neuroblasts and lKCs in the honey bee brain.

## Introduction

The European honey bee (*Apis mellifera* L.) is a eusocial insect that lives in a colony, and its workers shift their labors from nursing the brood to foraging according to their age after eclosion^1,2^. Honey bees exhibit high learning and memory ability, and successful foragers perform the ‘waggle dance’ to inform their nestmates of the location of a food source, which they memorize during foraging flights^1–3^. Due to their high learning and memory abilities, honey bees are extensively studied as a model for evaluating the mechanisms of learning and memory^4–10^. The molecular and neural bases underlying the high learning and memory ability of the honey bee, however, remain elusive.

In the honey bee brain, the mushroom bodies (MBs), a higher order center in the insect brain, comprise four types of interneurons, termed Kenyon cells (KCs): class I large-type KCs (lKCs), middle-type KCs, small-type KCs, and class II KCs, which exhibit distinct gene expression profiles [^11–13^, for review see^14–16^] and are classified according to the location and size of the somata^13,17,18^. The lKCs show preferential expression of five genes encoding proteins involved in Ca^2+^-signaling, such as Ca^2+^/calmodulin-dependent protein kinase II (CaMKII)^19^, which plays a central role in the synaptic plasticity that serves as a platform for learning and memory processes in many animal species^20–23^. Phosphorylated (activated) CaMKII is present in lKCs, but not in sKCs or class II KCs^24^. Findings from experiments using RNA interference and pharmacologic inhibition indicate that CaMKII is necessary for long-term memory^25,26^. Together, these characteristics of lKCs suggest their involvement in Ca^2+^-signaling-mediated learning and memory in the honey bee.

In addition to these genes involved in Ca^2+^-signaling, two genes encoding the transcription factors *Mblk-1* (*Mushroom body/large-type Kenyon-cell specific protein-1*) and *BR-C* (*Broad complex*) are also expressed in a lKC-preferential manner in pupal and adult worker brains^12,27–29^. *Mblk-1* was originally identified as a gene preferentially expressed in the MBs of the worker honey bee brain^12^. An *in vitro* reporter assay revealed that Mblk-1 is a sequence-specific transcription activator and phosphorylation at Ser444 by the Ras/MAPK pathway activates the transcriptional activity of Mblk-1^28,29^. Thus, Mblk-1 appears to play a role in regulating transcription downstream of the Ras/MAPK pathway in the lKCs.

Mblk-1 homologues are conserved beyond animal species^12,30–37^. E93, the *Drosophila* orthologue of honey bee Mblk-1 functions in morphogenesis during metamorphosis downstream of ecdysone-signaling *via* the ecdysone receptor as an ecdysteroid-regulated protein^30,38–40^. In contrast, MBR-1, the *Caenorhabditis elegans* orthologue of honey bee Mblk-1, is required for pruning excessive neurites as well as for learning and memory^32,41,42^. It is thus plausible that honey bee Mblk-1/E93 also functions in morphogenesis during metamorphosis downstream of ecdysone-signaling and/or in learning and memory at the adult stages. Although it is intriguing to speculate that Mblk-1 has roles in both pupal and adult brains, and that phosphorylation regulates its transcriptional activity in some way, the distribution and phosphorylation of Mblk-1 protein in pupal and worker honey bees have not yet been examined.

In the present study, we prepared affinity-purified antibodies against a partial recombinant Mblk-1 protein and a phosphorylated Mblk-1 (p-Mblk-1) peptide, and performed immunoblotting and immunofluorescence studies to investigate the distribution and phosphorylation status of Mblk-1 protein in both pupal and adult worker brains. Our findings suggest that Mblk-1 functions in the lKCs in both pupal and adult honey bee brains, and that p-Mblk-1 has pupal stage-specific functions in the MB neuroblasts and lKCs in the honey bee brain.

## Methods

### Animals

European honey bee (*Apis mellifera* L.) colonies maintained at The University of Tokyo (Faculty of Science Bldg 2, Hongo campus) were used. Some colonies were also purchased from a local dealer (Kumagaya Honeybee Farm, Saitama, Japan). Nurse bees and foragers were collected according to their behaviors and the degree of hypopharyngeal gland development, as described previously^43^. Briefly, workers that repeatedly inserted their heads into larval cells to feed the brood and had well-developed hypopharyngeal glands (i.e., glands that synthesize and secrete major royal jelly proteins) were collected as nurse bees. Workers that returned to their hives with pollen loads on their hind legs and had shrunken hypopharyngeal glands were collected as foragers. Re-orienting bees were collected essentially as described previously^44^. Briefly, after the hive entrance was closed, the location of the hive was changed at night. In the morning of the next day, re-orienting bees were collected as workers that exhibited orientation flight 15–20 min after the hive entrance was opened.

### Preparation of recombinant Mblk-1 fragments A and B and antibodies against fragments A and B

Mblk-1 cDNA fragments corresponding to fragment A (1272 bp, +1400 to +2671) and fragment B (1296 bp, +2576 to +3871) were amplified by polymerase chain reaction (PCR) with gene-specific primers and inserted into a pEThT vector to produce N-terminally His-tagged protein^28^. The expression construct was used to transform *Escherichia coli* BL21 (DE3). Expression of the recombinant fragment A/B proteins with a His tag at the N-terminus was induced by incubating transformed *E. coli* BL21(DE3) in medium containing 0.1 mM isopropyl β-D-1-thiogalactopyranoside (IPTG) at 37 °C for 4 h. The cells were then collected by centrifugation and lysed with lysis buffer [20 mM Tris-HCl, pH 7.5, containing 1% Triton X-100 and Complete EDTA free protease inhibitor cocktail (Roche, Japan)]. After inclusion bodies were isolated by centrifugation, the proteins were dissolved in 8 M urea buffer and purified in a denatured condition using Ni-NTA Agarose (Qiagen) according to the manufacturer’s protocol. The purified proteins eluted with 4 M urea buffer containing 150 mM imidazole were used to immunize rabbits and anti-fragment A/B antisera were obtained after five booster injections. Anti-fragment A/B antibodies were affinity-purified and each antibody was preabsorbed with the other fragment to eliminate cross-reactivity against both fragments. The antibody concentration was estimated based on the absorbance at 280 nm.

### Preparation of antibody against phosphorylated Mblk-1 peptide

Oligopeptides corresponding to amino acid positions 439 to 448 of Mblk-1 protein containing the MAPK phosphorylation site S444^29^ were chemically synthesized by Eurofins Genomics K.K. (Tokyo, Japan), either with or without phosphorylation of S444 (DQPPL[pS]PQSD and DQPPLSPQSD, respectively). Antibody against the phosphorylated peptide was affinity-purified from the antiserum raised against the phosphorylated peptide using the phosphorylated peptides and then preabsorbed with the non-phosphorylated peptides to obtain the purified antibody specific to the phosphorylated peptide. The specific reactivity of the obtained antibody was confirmed by enzyme-linked immunosorbent assay (ELISA) using the phosphorylated and non-phosphorylated peptides (Fig. S1). Preparation of the antibody and ELISA were performed by Eurofins Genomics K.K.

### Expression of full-length recombinant Mblk-1 in *E. coli*

The full-length Mblk-1 coding sequence (4794 bp) was amplified by PCR with gene-specific primers, inserted into a pEThT vector, and the sequence for 3x Hemagglutinin (HA) tag amplified by PCR from Mblk-1/pPac-PL^29^ was attached to the C-terminus sequence of Mblk-1 coding sequence. The resulting plasmid vector (Mblk-1-HA/pEThT) was used to transform *E. coli* BL21 (DE3). After the expression of 3x HA-tagged full-length recombinant Mblk-1 was induced by adding IPTG in Luria Bertani broth (2 ml), transformant *E. coli* cells were harvested by centrifugation and lysed with sodium dodecyl sulfate (SDS) sample buffer (62.5 mM Tris-HCl, pH 6.8, containing 2% SDS, 5% sucrose, and 0.005% bromophenol blue) for immunoblotting.

### Expression of full-length recombinant Mblk-1 in *Drosophila* SL-2 cells

SL-2 cells (Schneider’s Line 2 cells derived from *D. melanogaster* embryos)^45^ were maintained in Schneider’s *Drosophila* medium (Invitrogen) with the addition of heat-inactivated fetal bovine serum (MilliporeSigma) and antibiotics (50 U/ml penicillin G and 0.05 mg/ml streptomycin) (Invitrogen). The cells were grown in monolayers at 27 °C. We previously subcloned the full-length Mblk-1 cDNA with 3xHA tag sequence into the multicloning site (*Bam*HI-*Spe*I) of the *actin 5C* expression vector (pPac-PL)^46^, which we termed Mblk-1/pPac-PL^29^. Transfection was performed essentially as described previously^29^. SL-2 cells (0.8 × 10^6^ cells/ml) were cultured in 2 ml Schneider’s *Drosophila* medium containing heat-inactivated fetal bovine serum in a 12-well plate for 12 h at 27 °C to allow them to adhere to the dish, and the medium was discarded. *Drosophila* serum-free medium (2 ml) was added to the dish. Plasmid DNA (1 μg) was incubated with 8 μl Cellfectin II Reagent (Invitrogen) in 0.2 ml *Drosophila* serum-free medium (Invitrogen) for 10 min. The resulting mixture was added to the adhered cells and incubated for 4 h to accomplish transfection. The medium was then replaced with fresh Schneider’s *Drosophila* medium containing heat-inactivated fetal bovine serum and incubation was continued. Two days later, the number of cells was counted. The cells were harvested by centrifugation and lysed with lysis buffer (50 mM Tris-HCl, pH 7.4, containing 4% SDS and 150 mM NaCl) for immunoblotting.

### Dephosphorylation treatment

Brains were dissected from the heads of 5 worker pupae under binocular microscopy and homogenized in 150 µl buffered insect saline [20 mM Tris-HCl (pH 7.4), 130 mM NaCl, 5 mM KCl, 1 mM CaCl_2_, containing protease inhibitor cocktail (Roche)]. 2 ul (800 Unit) of Lambda Phosphatase (Santa Cruz Biotechnology, USA), 10 µl of 10x Lambda Phosphatase buffer and 10 µl of 10x MnCl_2_ solution (Santa Cruz Biotechnology) were added to the 65 µl homogenate and total volume was made up to 100 µl with water. The sample was incubated at room temperature for 30 min. As a negative control, in which dephosphorylation was inhibited, 10 µl of 10x PhosSTOP (Roche) was added instead of Lambda Phosphatase. After the incubation, the samples were used for immunoblotting.

### Immunoblotting

After each 10 worker pupae, nurse bees, re-orienting bees, and foragers were anesthetized on ice, their brains were dissected from the heads under binocular microscopy. Heads without brains, thoraxes, and abdomens from three individuals were also dissected and homogenized in 250 µl buffered insect saline containing protease inhibitor cocktail (Roche) and PhosSTOP (Roche). The lysate of *E. coli* cells and SL-2 cells expressing full length Mblk-1 was prepared as described above. The protein concentration was determined using a BCA Protein Assay Reagent Kit (Thermo Scientific Pierce), and the same amount of protein was subjected to SDS-polyacrylamide gel electrophoresis (PAGE). Immunoblotting was performed essentially as described previously^47^ using 2.0 µg/ml each of anti-fragment A antibody, anti-p-Mblk-1 antibody, normal rabbit IgG (MBL) as a negative control, anti-HA high affinity from rat IgG_1_ (MilliporeSigma), or beta-actin (C4) mouse monoclonal antibody (sc-47778, Santa Cruz Biotechnology) as the primary antibody, and 0.1 µg/ml horse radish peroxidase-conjugated anti-rabbit IgG antibody (MilliporeSigma), anti-rat IgG (whole molecule)-Peroxidase antibody produced in rabbit (MilliporeSigma), or HRP goat anti-mouse IgG antibody (KPL) as the secondary antibody. For blocking, Tris-Buffered Saline with Tween 20 (TBST; 50 mM Tris-HCl (pH 7.4), 138 mM NaCl, 2.7 mM KCl, 0.05% Tween20) containing 5% Skim Milk Powder (Wako) was used in all immunoblotting experiments except for that of Lambda phosphatase treated samples, and TBST containing 5% ECL Prime Blocking Reagent (GE Healthcare Life Sciences) and 4% bovine serum albumin (BSA) was used for the immunoblotting experiment of Lambda phosphatase treated samples. After the membrane was treated with ECL Select Western Blotting Detection Reagent (GE Healthcare Life Sciences), the antigen was detected by chemiluminescence using ImageQuant LAS 4000mini (GE Healthcare Life Sciences). To perform immunoblotting with different antibodies on the same membrane (re-probing), the membranes were incubated at 50 °C for 30 min in stripping buffer (62.5 mM Tris-HCl pH 6.7 at 50 °C, 2% SDS, 100 mM β-mercaptoethanol) to remove the bound antibodies. After washing two times with TBST at room temperature for 10 min, the membranes were processed for blocking.

### Immunohistochemistry

Brains were dissected from the heads of three individual adult workers and heads were dissected from three individual pupal workers. The adult worker brains and pupal heads were embedded in OCT compound (SAKURA Tissue-Tek) and rapidly frozen on dry ice. The adult worker brains and pupal heads were then cut into 10-µm serial sections using a cryostat (Leica  CM1850) and fixed in phosphate buffered saline (PBS; 137 mM NaCl, 2.7 mM KCl, 10 mM Na_2_HPO_4_, 1.8 mM KH_2_PO_4_) containing 4% paraformaldehyde and Triton X-100 (0.1%) at room temperature for 40 min. After washing the brain sections in PBS-Tx (PBS containing 0.1%  or 0.2% Triton X-100) and blocking them in 2% BSA/PBS-Tx [PBS-Tx(0.1%) containing 2% BSA] at room temperature for 1 h, they were incubated with 2% BSA/PBS-Tx containing 0.50 µg/ml each of affinity-purified anti-fragment A antibody, anti-p-Mblk-1 antibody, or normal rabbit IgG as a negative control. Next, the brain sections were washed three times with PBS-Tx and incubated with 4.0 µg/ml Alexa Fluor 488 anti-rabbit IgG antibody for 1 h. The brain sections were again washed three times with PBS, stained with 4′,6-diamidino-2-phenylindole, and observed by fluorescence microscopy (ZEISS Axio Imager Z1) and confocal microscopy (ZEISS LSM710). The brightness and contrast of images for each signal were adjusted and the images were merged using ImageJ 1.50i.

## Results

### Immunoblotting of Mblk-1 in the worker body parts

**Figure 1:**
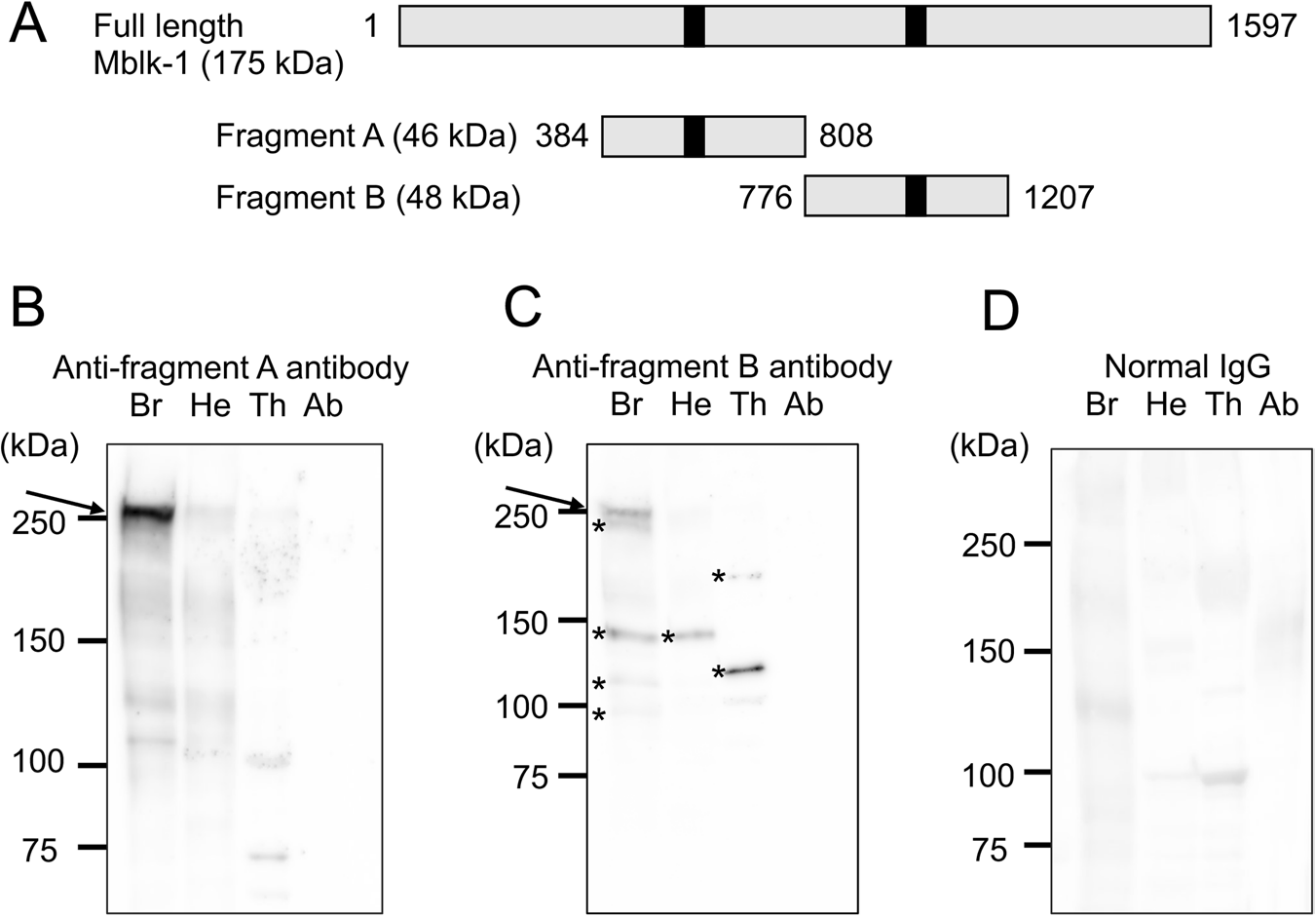
Immunoblotting analysis of Mblk-1 in various adult body parts using anti-fragment A and B antibodies. (**A**) Schematic drawing of fragments A and B, against which the antibodies were prepared. The grey bars indicate the primary structures of full-length Mblk-1 and fragments A and B. Black boxes indicate two DNA-binding domains of Mblk-1. The numbers indicate amino acid positions. (**B–D**) Immunoblotting analysis of worker brains (Br), heads without brains (He), thoraxes (Th), and abdomens (Ab) using the anti-fragment A antibody **(B**), anti-fragment B antibody (**C**), and normal IgG as a negative control (**D**). The positions of Mblk-1 are indicated by arrows in panels (B,C). Asterisks in panel (C) indicate putative degradation products of Mblk-1 or unrelated proteins, which were immunocrossreactive with Mblk-1. Note that the raw data, containing the entire signals, which were obtained by using the blotted membrane and anti-fragment A antibody [Fig. S2(A) panel] or by re-probing the same membrane with normal IgG [Fig. S2(C) panel], are cropped and grouped as Fig. 1 panels (B,D), respectively, and that the raw datum containing the entire signals, which was obtained by re-probing the same membrane, which had been used for Fig. 1 panels (B,D), with anti-fragment B antibody [Fig. S2(B) panel], is cropped and grouped as Fig. 1 panel (C). The exposure times for all of panels (B) to (D) were 30 sec.

As the Mblk-1 protein (approximately 175 kDa) is too long to efficiently express the entire recombinant Mblk-1 protein in *E. coli*, we synthesized two truncated recombinant Mblk-1 proteins; fragment A corresponding to amino acid positions 384–808 (~46 kDa), and fragment B corresponding to amino acid positions 776–1207 (~48 kDa) of Mblk-1, each of which contains a conserved putative DNA binding site (Fig. 1A)^28^. We raised antibodies against each of the recombinant fragments A and B, and affinity-purified the antibodies (hereafter, these antibodies are termed anti-fragment A and anti-fragment B antibodies, respectively). If bands with the same molecular weights were detected with the anti-fragment A and B antibodies, respectively, in our immunoblotting experiments, the band was assumed to correspond to Mblk-1.

Immunoblotting using the anti-fragment A antibody and the homogenates of brains, heads without brains, thoraxes, and abdomens of workers that were randomly caught inside a hive detected a major band with molecular weight of approximately 250 kDa only in the brain sample, but scarcely detected any discrete bands in the heads without brain, thorax, or abdomen samples (Fig. 1B). On the other hand, immunoblotting using the anti-fragment B antibody not only detected a major band with a molecular weight of approximately 250 kDa in the brain sample, but also detected some other discrete bands in the brain (bands with approximate molecular weights of 240, 145, 120, and 95 kDa), heads without brain (a band with an approximate molecular weight of 145 kDa), and thorax samples (bands with approximate molecular weights of 190, and 125 kDa; Fig. 1C). No significant bands were detected in a control experiment using normal IgG as the primary antibody, indicating that the above-described bands were specifically detected by anti-fragment A/B antibodies (Fig. 1D). We confirmed that the positions for the 250-kDa bands detected by anti-fragment A/B antibodies were the same by re-probing the same membrane used for the immunostaining with anti-fragment A antibody, with anti-fragment B antibody (data not shown).

### Immunoblotting of HA-tagged Mblk-1 expressed in *E. coli* and *Drosophila* SL-2 cells

We assumed that the 250-kDa bands, which were commonly detected with the anti-fragment A/B antibodies in brain samples, corresponded to Mblk-1, and that the other bands detected in the brain, head without brain, thorax, and abdomen samples with anti-fragment B antibody corresponded to degraded Mblk-1 proteins lacking the fragment A region or unrelated proteins that were immunocrossreactive with the anti-fragment B antibody (Fig. 1C). The molecular weight (250 kDa) of the commonly detected bands, however, was much greater than the predicted molecular weight (175 kDa) of Mblk-1. We hypothesized that endogenous Mblk-1 is somehow modified so that the molecular weight of the Mblk-1 detected by immunoblotting increases to 250 kDa.

**Figure 2:**
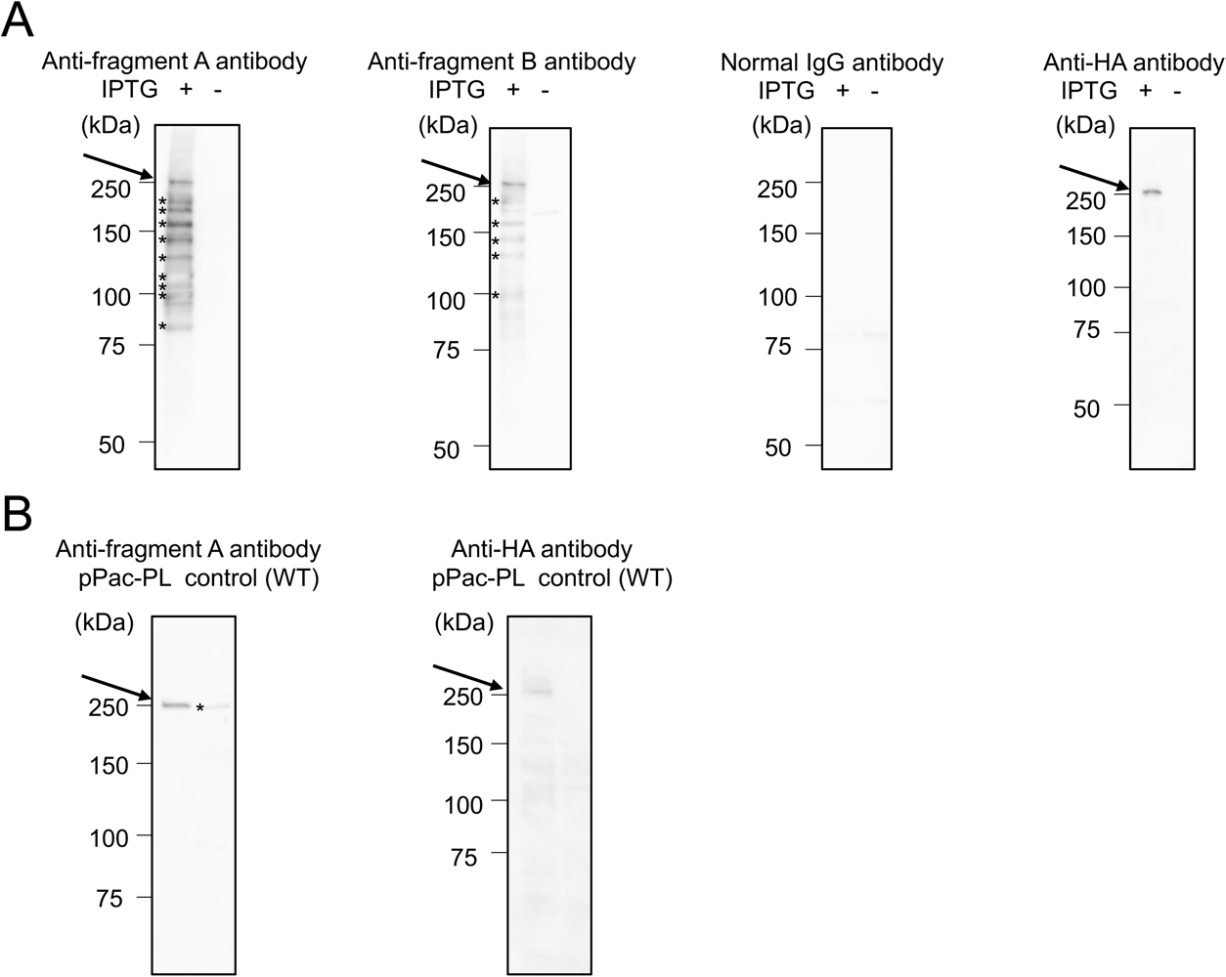
Immunoblotting analysis of Mblk-1 in *E. coli* and *Drosophila* SL-2 cells expressing HA-tagged full-length Mblk-1. (**A**) Immunoblotting analysis of lysate of *E. coli* expressing Mblk-1 and treated with (+) or without IPTG (−). The position of Mblk-1 is indicated by an arrow. Asterisks indicate putative degradation products or not fully translated proteins of Mblk-1. (**B**) Immunoblotting analysis of lysate of *Drosophila* SL-2 cells harboring a vector to overexpress Mblk-1 (pPac-PL) or wild type cells (control). The position of Mblk-1 is indicated by an arrow. Note that the raw data, containing the entire signals, which were obtained using different membranes and anti-fragment A antibody [Fig. S3(A) panel and Fig. S3(B) panel], are cropped and grouped as Fig. 2 panels (A,B), respectively. The exposure time for Fig. 2 panel (A) was 20 sec, 50 sec, 50 sec, and 100 sec, respectively from the left side. The exposure time for Fig. 2 panel (B) was 150 sec and 580 sec, respectively from the left side.

To test this notion, we performed immunoblotting using the lysate of *E. coli* expressing 3xHA-tagged full-length recombinant Mblk-1 protein, anti-fragment A antibody, anti-fragment B antibody, normal rabbit IgG as a negative control, and anti-HA tag antibody. Although many bands were detected in an IPTG induction-dependent manner in *E. coli* lysates, the molecular weight of the largest band was approximately 250 kDa (Fig. 2A), which coincided with the results of immunoblotting with anti-fragment A/B antibodies and the worker brain samples (Fig. 1B,C). In contrast, only a single 250-kDa band was detected with anti-HA tag antibody in the lysate of *E. coli* expressing 3x HA-tagged full-length recombinant Mblk-1 protein, clearly indicating that the 250-kDa protein detected in the worker brain lysate (Fig. 1B,C) and in the *E. coli* lysate (Fig. 2A) with anti-fragment A/B antibody corresponded to the Mblk-1 protein. We also performed immunoblotting using the lysate of *Drosophila* SL-2 cells expressing 3xHA-tagged full-length recombinant Mblk-1 protein, anti-fragment A antibody, and anti-HA tag antibody. In this case, a discrete 250-kDa band was detected in the lysate of cells expressing 3xHA-tagged full-length Mblk-1 (Fig. 2B). Notably, a ~250-kDa weak band was detected in the lysate of wild type cells with anti-fragment A antibody (Fig. 2B). It is plausible that the ~250-kDa weak band detected in the wild type cell lysate represents endogenous *Drosophila* E93, although its predicted molecular weight is approximately 150 kDa^38^.

On the basis of these results, we concluded that the 250-kDa band detected in the *E. coli* lysate and SL-2 cell lysate corresponded to the intact Mblk-1 protein, and many other bands whose molecular weights were smaller than 250 kDa detected in the *E. coli* lysate corresponded to degraded fragments or not fully translated fragments of the Mblk-1 protein, because HA tag was fused to the C terminus of Mblk-1 (Fig. 2A). It is plausible that the Mblk-1 protein is fragile and thus easily degraded in honey bee homogenates. Taken together, we concluded that Mblk-1 was mainly expressed in the brains, and scarcely expressed in the heads without heads, thoraxes, and abdomens of the worker honey bee. We also decided to use the anti-fragment A antibody, which detected Mblk-1 (250-kDa band) as a major band in the worker brain, to analyze the distribution and localization of the Mblk-1 protein hereafter.

### Developmental stage- and/or worker labor-dependent expression of Mblk-1

**Figure 3:**
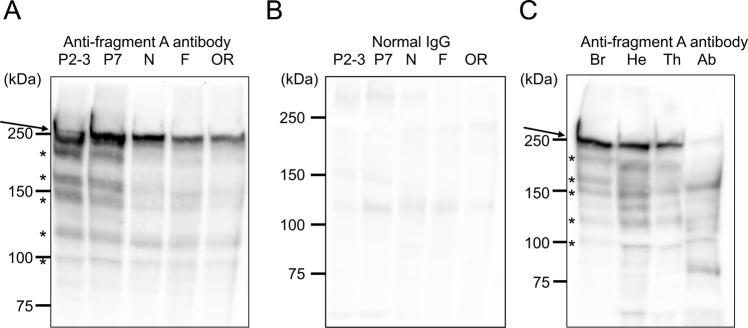
Immunoblotting analysis of developmental stage- and/or worker labor-dependent expression of Mblk-1. (**A,B**) Immunoblotting analysis of Mblk-1 in the brain lysate of P2-3 (P2-3) and P7 pupae (P7), nurse bees (N), foragers (F), and re-orienting bees (OR) using anti-fragment A antibody (A) and normal IgG as a control (**B**). (**C**) Immunoblotting analysis of pupal brains (Br), heads without brains (He), thoraxes (Th), and abdomens (Ab) using anti-fragment A antibody. The positions of Mblk-1 are indicated by arrows in panels (A,C). Asterisks in panels (A,C) indicate putative degradation products of Mblk-1. Note that the raw data, containing the entire signals, which were obtained by using the blotted membrane and anti-fragment A antibody [Fig. S4(A) panel] or by re-probing the same membrane with normal IgG [Fig. S4(B) panel], are cropped and grouped as Fig. 3 panels (A,B), respectively, and that the raw datum containing the entire signals, obtained using a different membrane and anti-fragment A antibody [Fig. S4(C) panel], is cropped and grouped as Fig. 3 panel (C). The exposure times for all of panels (A) to (C) were 30 sec.

We next performed immunoblotting with the anti-fragment A antibody to examine whether the expression of Mblk-1 changes according to the developmental stage and/or division of labor of workers. We analyzed nurse bees, foragers, and re-orienting bees because we expected that Mblk-1 plays more important roles in foragers and/or re-orienting bees, which seem to require higher learning and memory abilities. Although the 250-kDa band was detected in the brains of pupae (P2-3 and P7) and all examined adults (nurse bees, foragers, and re-orienting bees), the intensity of the 250-kDa band was stronger in the pupal stages than in the adult stages (Fig. 3A). Again, many other faint bands whose molecular weights were smaller than 250 kDa and seemed to correspond to degraded fragments of the Mblk-1 protein were detected in all samples, especially in pupal samples (Fig. 3A). No noteworthy bands were detected in a control experiment with normal IgG, indicating that the above-described bands were specifically detected by the anti-fragment A antibody (Fig. 3B). We also performed immunoblotting using various pupal worker body parts. The 250-kDa bands were detected as major bands in the brain and head without brain, thorax samples, and as a minor band in abdomen samples (Fig. 3C). Taken together, our results suggest that, while Mblk-1 functions mainly in the brain at adult stage, it functions not only in the brain but also in parts of head other than brain and thorax at pupal stages.

### Developmental stage- and/or body part-preferential phosphorylation of Mblk-1

**Figure 4:**
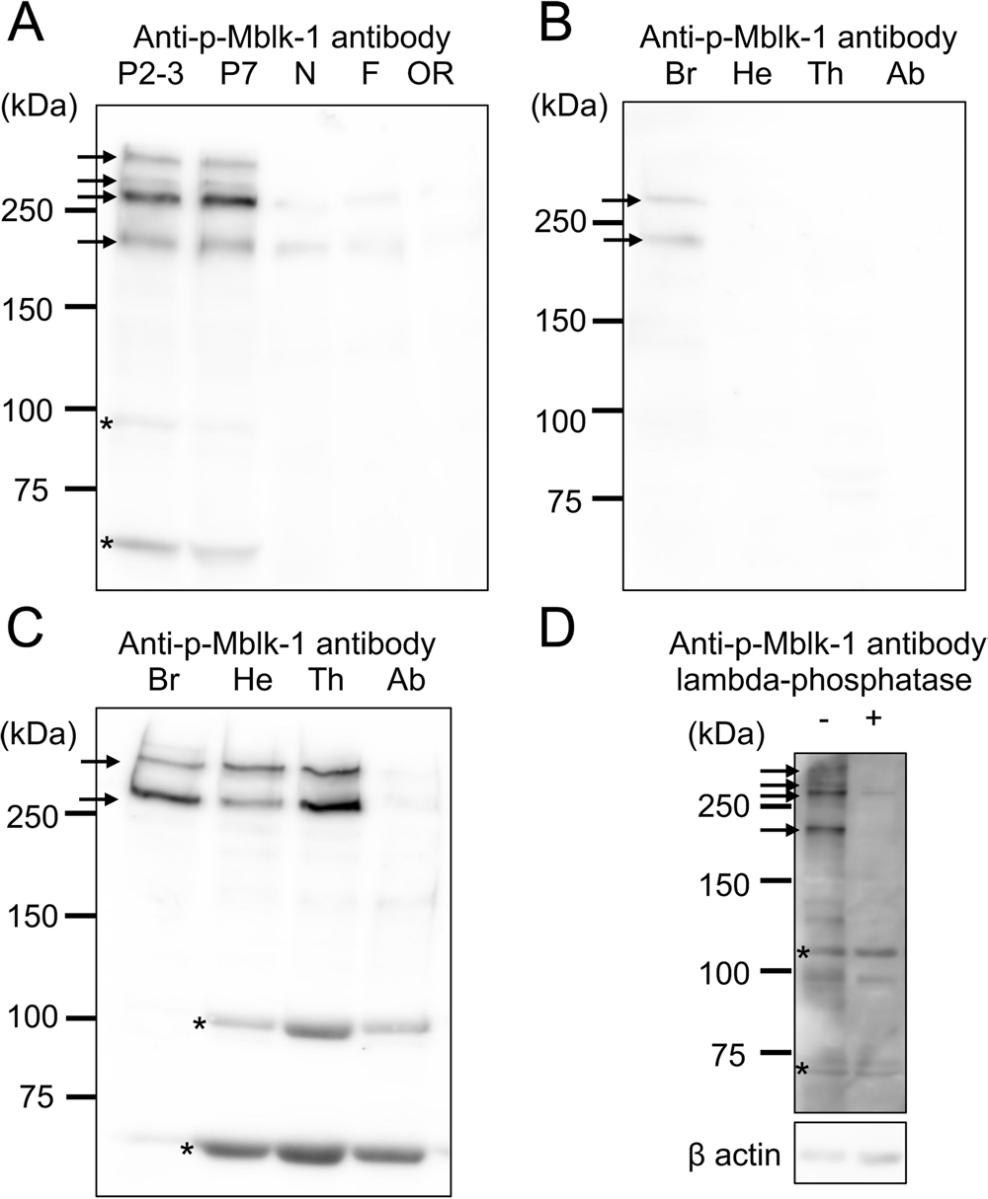
Immunoblotting analysis of developmental stage- and/or worker labor-dependent expression of p-Mblk-1. (**A**) Immunoblotting analysis of p-Mblk-1 in the brain lysate of P2-3 (P2-3) and P7 pupae (P7), nurse bees (N), foragers (F), and re-orienting bees (OR). (**B**) Immunoblotting analysis of p-Mblk-1 in adult worker brains (Br), heads without brains (He), thoraxes (Th), and abdomens (Ab) using anti-p-Mblk-1 antibody. (**C**) Immunoblotting analysis of p-Mblk-1 in pupal brains (Br), heads without brains (He), thoraxes (Th), and abdomens (Ab) using anti-p-Mblk-1 antibody. (**D**) Immunoblotting analysis using anti-p-Mblk-1 antibody and the pupal worker brain homogenate subjected to dephosphorylation treatment. Using the same membrane, the amount of applied protein was estimated with beta-actin (C4) antibody. The positions of the four forms of p-Mblk-1 are indicated by arrows in panels (A–D). Asterisks in panels (A,C,D) indicate putative degradation products of Mblk-1, or unrelated proteins, which were immunocrossreactive with Mblk-1. Note that the raw data, containing the entire signals, which were obtained by using different membranes and anti-p-Mblk-1 antibody [Fig. S5(A–D) panels], are cropped and grouped as Fig. 4 panels (A) to (D), respectively. The exposure times for panels (A) to (C) were 30 sec, and the ones for panels (D, p-Mblk-1) and (D, β actin) were 40 and 60 sec respectively.

We next examined stage- and/or body part-preferential phosphorylation of Mblk-1. For this, we raised an antiserum against a synthetic oligopeptide that corresponded to amino acid positions 439 to 448 with a phosphorylated Ser444 residue^29,48^. The antibody was affinity-purified using the above phosphorylated peptide and then preabsorbed using non-phosphorylated peptide (hereafter, this antibody is termed anti-p-Mblk-1 antibody). The anti-p-Mblk-1 antibody was expected to selectively recognize p-Mblk-1, but not non-phosphorylated Mblk-1. We first performed immunoblotting using the anti-p-Mblk-1 antibody and homogenates of brains of pupae (P2-3 and P7), nurse bees, foragers, and re-orienting bees, to analyze developmental stage-preferential phosphorylation of Mblk-1. Six bands of approximately 300, 270, 260, 210, 100, and 50 kDa were detected in pupal brains (P2-3 and P7), whereas only faint bands of approximately 260 and 210 kDa were detected in adult worker (nurse bees, foragers, and re-orienting bees) brains (Fig. 4A). No noteworthy bands were detected in a control experiment with normal IgG, indicating that the above-described bands were specifically detected by the anti-p-Mblk-1 antibody (Fig. S6).

We then analyzed body part-preferential phosphorylation of Mblk-1 using the anti-p-Mblk-1 antibody and the homogenates of brains, heads without brains, thoraxes, and abdomens of adult workers. Two faint bands corresponding to 260 and 210 kDa were detected in only the brain homogenate, and not in the homogenates of heads without brains, thoraxes, and abdomens (Fig. 4B). Analysis of body part-preferential phosphorylation of Mblk-1 using the anti-p-Mblk-1 antibody and homogenates of brains, heads without brains, thoraxes, and abdomens of early pupae (P2-3 stages) revealed two bands of approximately 300 and 260 kDa mainly in the homogenates of brains, heads without brains, and thoraxes (Fig. 4C). In addition, two bands of approximately 100 and 50 kDa were mainly detected in the homogenates of heads without brains, thoraxes, and abdomens (Fig. 4C). No noteworthy bands were detected in a control experiment with normal IgG, indicating that the above-described bands were specifically detected by the anti-p-Mblk-1 antibody (Fig. S6). These results indicate that four forms (300-, 270-, 260-, and 210-kDa bands) of p-Mblk-1 are expressed in some pupal body parts (brains, head without brains and thoraxes), but are scarcely expressed in the adult body. The bands of approximately 100 and 50 kDa may represent unrelated but immunocrossreactive proteins, because their distributions among body parts were inconsistent with those of Mblk-1 detected in Fig. 3C.

To further investigate whether the anti-p-Mblk-1 antibody actually recognizes phosphorylated Mblk-1 proteins, we performed immunoblotting using anti-p-Mblk-1 antibody and pupal worker brain homogenate, which had been subjected to dephosphorylation treatment with lambda protein phosphatase. Compared to the non-treated (negative control) sample, the densities of 300-, 270-, 260-, and 210-kDa bands were significantly reduced, whereas the densities of 100- and 50-kDa bands did not differ significantly, strongly suggesting that the 300-, 270-, 260- and 210-kDa bands corresponded to phosphorylated Mblk-1 proteins, whereas both 100-kDa and 50-kDa proteins represented unrelated but immonocrossreactive proteins. It is noteworthy that the immunoreactivity of the approximately 250 kDa bands (260 and 270 kDa bands) weakened after incubation with lambda phosphatase, and therefore, it is plausible that the anti-p-Mblk-1 antibody either did not recognize non-phosphorylated Mblk-1, or had very weak immunocrossreactivity, if any (Fig. 4D).

### Immunohistochemistry of Mblk-1 and phosphorylated Mblk-1

**Figure 5:**
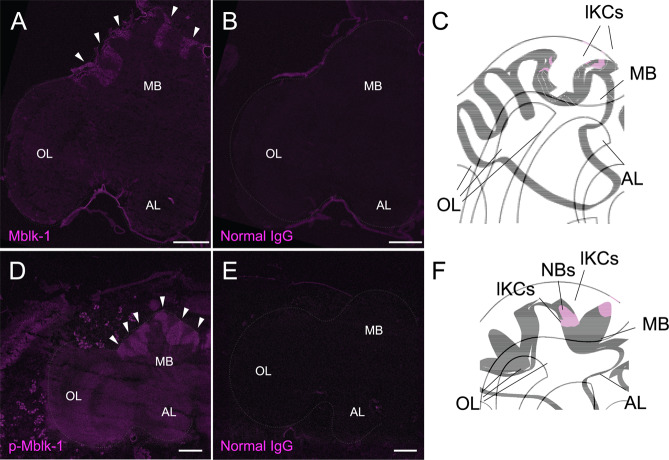
Immunohistochemistry of Mblk-1 and p-Mblk-1. (**A**) Immunohistochemistry of Mblk-1 in the brain of an adult worker bee. Signals in large-type KCs (lKC) are indicated by arrowheads. (**B**) Immunohistochemistry using normal rabbit IgG in the brain of an adult worker. (**C**) Schematic drawing of the left hemisphere of the brain observed in panels (A,B). (**D**) Immunohistochemistry of p-Mblk-1 in the brain of an early pupal worker. (**E**) Immunohistochemistry using normal rabbit IgG in the brain of an early pupal worker. (**F**) Schematic drawing of the left hemisphere of the brain observed in panels (D,E). Signals are indicated by arrowheads in panels (A,D). MB, mushroom bodies; OL, optic lobes; AL, antennal lobes; lKCs, large-type KCs; NBs, neuroblasts. Bars indicate 200 μm. Images were captured on the same condition with Tile Scan and combined into a single image in the software ZEN 2009.

**Figure 6:**
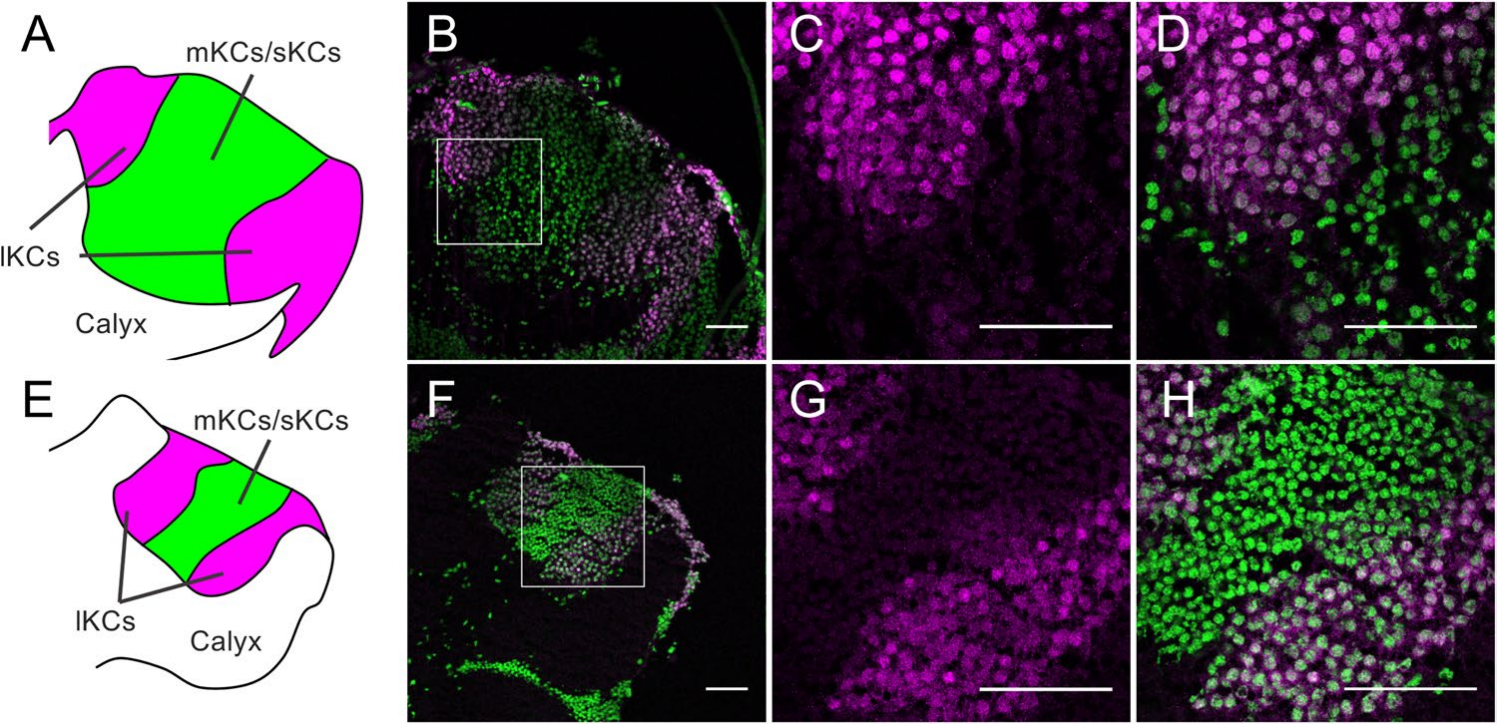
Immunohistochemistry of Mblk-1 in the brains of a pupa and a re-orienting bee. (**A–H**) Immunohistochemistry of Mblk-1 in the brains of a pupa (**A–D**) and a re-orienting bee (**E–H**). (**A,E**) Schematic drawings of the MB calyces shown in panels (B,F), respectively. lKCs, large-type KCs; mKCs, middle-type KCs; sKCs, small-type KCs. (**B,F**) Immunohistochemistry of Mblk-1 in the brains of a pupa (**B**) and a re-orienting bee (**F**). Signals for fluorescence of Mblk-1 and DAPI are shown in magenta and green, respectively. (**C**,**D**,**G**,**H**) Magnified views of boxed area shown in panels (B,F), respectively. Bars indicate 50 μm.

Some transcription factors, such as Ikaros^49^ and SRY-related HMG-box 11 (SOX11)^50^, translocate from the cytoplasm to the nuclei upon activation by phosphorylation. We therefore performed an immunofluorescence study using the anti-fragment A antibody, anti-p-Mblk-1 antibody, normal IgG (as a negative control) and brain sections of pupal and adult workers that were randomly collected from the hives. Anti-fragment A antibody specific Mblk-1-like immunoreactivity was detected preferentially in the KC somata in the MBs of the worker brain (Fig. 5A,B), which is consistent with our previous *in situ* hybridization results that Mblk-1 mRNA is expressed preferentially in the MBs of the worker brain^12^. The somata of Mblk-1-like immunoreactive KCs localized at the outer edges inside the MB calyces in both pupal and adult worker brains (Figs. 5 and 6). In addition, anti-p-Mblk-1 antibody specific p-Mblk-1-like immunoreactivity was also preferentially detected in the somata of KCs in the MBs of the brains of an early pupal worker. The somata of p-Mblk-1-like immunoreactive KCs localized not only at inside edges but also at inner core of the MB calyces (Fig. 5D,E).

**Figure 7:**
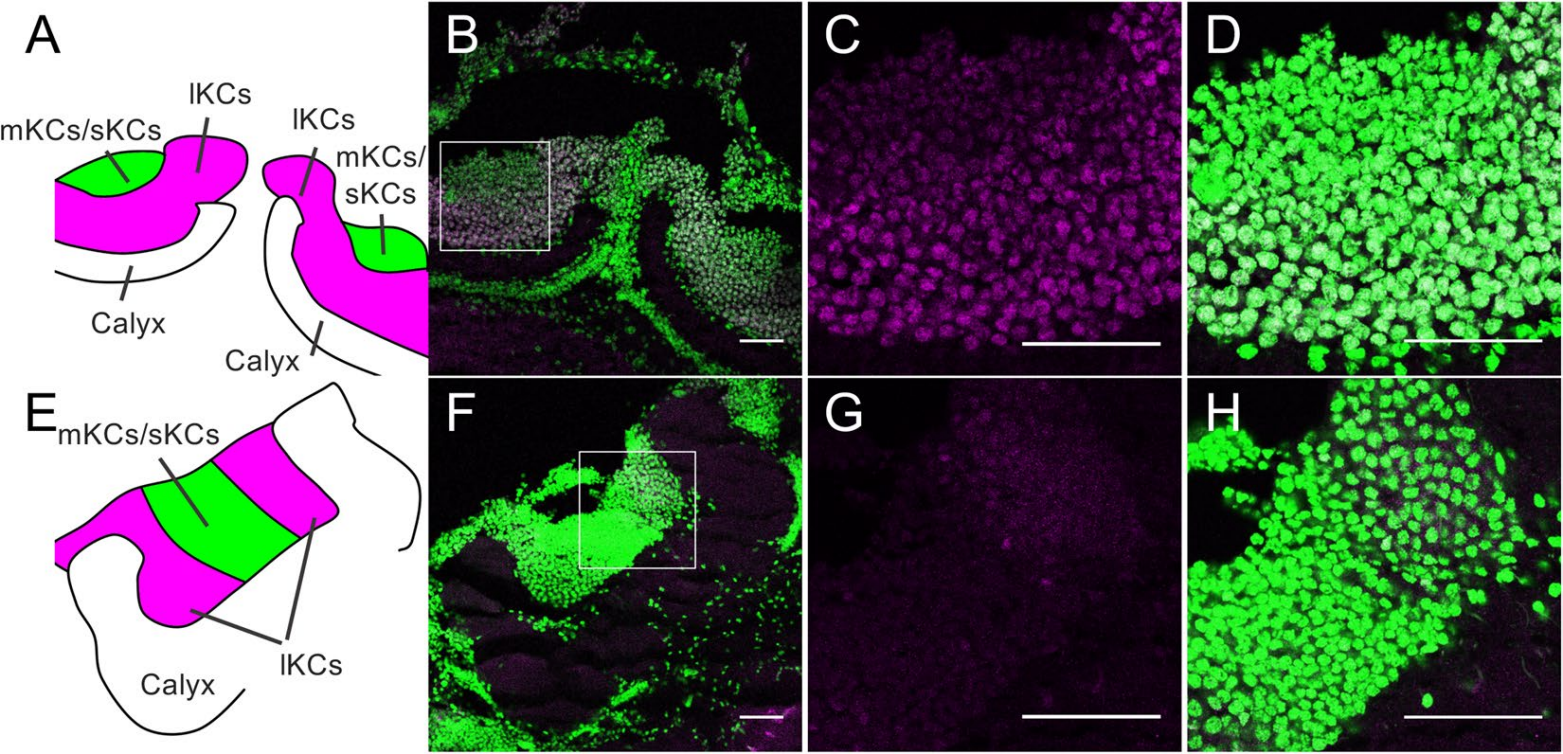
Immunohistochemistry of p-Mblk-1 in the brains of a pupa and a re-orienting bee. (**A–H**) Immunohistochemistry of p-Mblk-1 in the brains of a pupa (**A–D**) and a re-orienting bee (**E–H**). (**A,E**) Schematic drawings of the MB calyces shown in panels (B,F), respectively. lKCs, large-type KCs; mKCs, middle-type KCs; sKCs, small-type KCs. (**B,F**) Immunohistochemistry of p-Mblk-1 in the brains of a pupa (**B**) and a re-orienting bee (**F**). Signals for fluorescence of p-Mblk-1 and DAPI are shown in magenta and green, respectively. (**C,D,G,H**) Magnified views of boxed area shown in panels (**B,F**), respectively. Bars indicate 50 μm.

**Figure 8:**
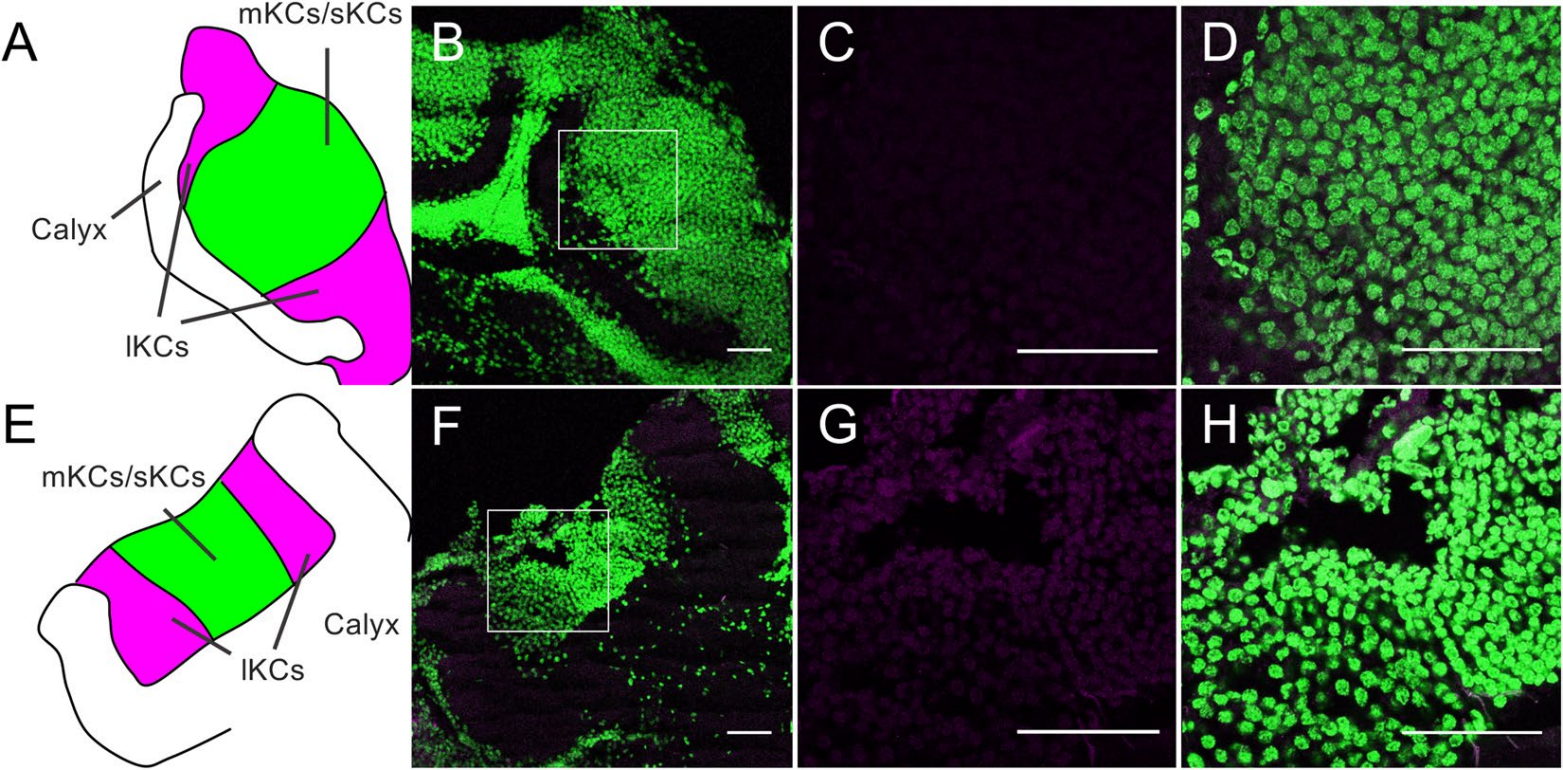
Immunohistochemistry of brain sections of a pupa and a re-orienting bee using normal IgG. (**A–H**) A control experiment using normal IgG for immunohistochemistry of p-Mblk-1 in the brain sections of a pupa (**A–D**) and a re-orienting bee (**E–H**). (**A,E**) Schematic drawings of the MB calyces shown in panels (B,F), respectively. lKCs, large-type KCs; mKCs, middle-type KCs; sKCs, small-type KCs. (**B,F**) A control experiment using normal IgG for immunohistochemistry of p-Mblk-1 in the brain sections of a pupa (**B**) and a re-orienting bee (**F**). Staining with normal IgG and DAPI are shown in magenta and green, respectively. (**C,D**,**G,H**) Magnified views of boxed area shown in panels (B) and (F), respectively. Bars indicate 50 μm.

We then performed an immunofluorescence study using the anti-fragment A, the anti-p-Mblk-1 antibody, and brain sections of pupal and adult (nurse bees, foragers, and re-orienting bees) workers to analyze the subcellular localization of Mblk-1 and p-Mblk-1 in the KCs of the MBs. Mblk-1-like immunoreactivity was again detected preferentially in the somata of KCs in the MBs in the pupal workers, nurse bees, foragers, and re-orienting bees (Figs. 6 and S7). Detailed examination revealed that Mblk-1 localized not only in the nuclei but also in the cytoplasm of the somata of KCs of pupae (Fig. 6A–D), re-orienting bees (Fig. 6E–H), nurse bees (Fig. S7A–D), and foragers (Fig. S7E–H). Similarly, p-Mblk-1-like immunoreactivities localized not only in the nuclei but also in the cytoplasm of the somata of KCs of pupae (Fig. 7A–D), re-orienting bees (Fig. 7E–H), nurse bees (Fig. S8A–D), and foragers (Fig. S8E–H), although fluorescence due to p-Mblk-1-like immunoreactivity in the KCs of the re-orienting bees, nurse bees, and foragers was very weak compared to that in pupae, which is consistent with our previous immunoblotting results (Fig. 4A). The somata of p-Mblk-1-like immunoreactive KCs localized at the inside edges of the MB calyces in pupal and adult worker brains (Figs. 7B,F and S8B,F). No noteworthy staining was detected in control experiments with normal IgG in pupal brains, indicating that the above-detected signals were specifically detected by the anti-p-Mblk-1 antibody (Fig. 8C). On the other hand, in adult brains, some signals were detected, but they were uniform in KCs and seem to be background signals (Fig. 8G). These findings suggest that both Mblk-1 and p-Mblk-1 are active to regulate transcription at least in some KCs throughout the pupal and adult stages.

## Discussion

Our immunoblotting analyses using the affinity-purified anti-fragment A and the anti-fragment B antibodies commonly detected a 250-kDa band only in the brains of adult worker body parts (Fig. 1), suggesting that Mblk-1 is preferentially expressed in the brains of adult workers. The molecular weight of the commonly detected band in immunoblotting using honeybee brain, however, was estimated to be approximately 250 kDa (Fig. 1), which was much larger than that estimated from the amino acid sequence of Mblk-1 (~175 kDa). Exogenous expression of full-length Mblk-1 protein resulted in the production of an immunoreactive band around 250 kDa in both bacteria and an insect cell line (Fig. 2), suggesting that full-length Mblk-1 protein likely migrates to the position of 250 kDa in SDS-polyacrylamide gel electrophoresis (PAGE) analysis. Immunoblotting of full-length Mblk-1 expressed in *E. coli* also suggested that the high molecular weight (250 kDa) of Mblk-1 cannot be attributed to a modification in eukaryotic cells. An unbalanced charge of a protein sometimes affects the mobility of the protein in SDS-PAGE^51,52^, but the isoelectric point of Mblk-1 is approximately 7.2 and thus overestimation of the molecular weight of Mblk-1 cannot be attributed to a biased amino acid composition of Mblk-1. In addition, the mobility of the Mblk-1 immunoreactive band (~250 kDa) did not change, even when brain lysate was treated with N-methylmaleimide, a reagent that modifies thiol residues in a protein, instead of β-mercaptoethanol, arguing against the possibility that Mblk-1 forms a dimer on the SDS-PAGE (data not shown). Thus, the reason for the overestimated molecular weight of Mblk-1 is currently unknown. We speculate that Mblk-1 denatured by SDS may have an abnormal three-dimensional structure due to its unique primary structure that results in low mobility on SDS-PAGE.

It is noteworthy that Mblk-1 is suggested to play a role in the adult worker brain, considering that Mblk-1/E93 functions in morphogenesis downstream of the ecdysone receptor during larval and pupal stages in *Drosophila*^30,36,38–40,53,54^. Immunoblotting using P2-3 (early) and P7 (late) pupae and nurse bees, foragers, and re-orienting bees, however, revealed that Mblk-1 was rather constitutively expressed throughout the pupal and adult stages, irrespective of the adult worker tasks (Fig. 3), suggesting that Mblk-1 functions in the adult brain as a fundamental component of the learning and memory processes, if any. Immunoblotting of various pupal body parts suggested that Mblk-1 functions in the morphogenesis of not only brains but also in parts of the head other than the brains and in thoraxes during the pupal stages. It is thus plausible that Mblk-1 restricts the organs where it functions associated with development through the pupal to adult stages, and specializes to function in the lKCs in the adult brain.

Immunoblotting using anti-p-Mblk-1-antibody revealed that four forms of p-Mblk-1 (300-, 270-, 260-, and 210-kDa bands), were strongly detected in the pupal brains, heads without brains and thoraxes but not in adult body parts, suggesting that p-Mblk-1 predominantly functions at the pupal stages (Fig. 4). It is possible that the major 260-kDa bands represent the phosphorylated form of full-length Mblk-1 (p-Mblk-1) and that the three sub-major bands at 300 kDa, 270 kDa, and 210 kDa, which exhibited similar developmental stage- and body-part preferential distribution patterns with 260-kDa band, correspond to p-Mblk-1 with further post-translational modifications and partly degraded p-Mblk-1, respectively. It is noteworthy that none of the four forms of p-Mblk-1 were detected by immunoblotting using the anti-fragment A antibody (Figs. 3A and 4A). These results suggest that each of the four forms of p-Mblk-1 represents a minor population of Mblk-1. In other words, only a limited population of Mblk-1 is phosphorylated in both the pupal and adult stages, implying that phosphorylation-mediated modulation of Mblk-1 may induce additional functions of Mblk-1 rather than entirely replace the function of the non-phosphorylated Mblk-1.

Finally, we used immunohistochemistry to analyze the subcellular localization of both Mblk-1 and p-Mblk-1 in pupal and adult worker brains. We originally hypothesized that p-Mblk-1 may represent an active form as a transcription factor and would be localized in the nuclei, whereas the non-phosphorylated form of Mblk-1 represents a non-active form as a transcription factor and would be localized mainly in the cytoplasm. Immunohistochemistry using the anti-fragment A antibody and brain sections of re-orienting bees revealed that Mblk-1-like immunoreactivity was localized almost exclusively in the somata of KCs located at the inside edges of MB calyces in the adult brains (Fig. 5A). Given that Mblk-1 mRNA is preferentially expressed in the lKCs in worker brain^12^, these Mblk-1-like immunoreactive KCs are likely to be lKCs. In contrast, p-Mblk-1-like immunoreactivity was localized not only in the lKCs, but also in the somata of cells located at the inner core inside the MB calyces in the brains of early pupae (Fig. 5D). Since MB neuroblasts are known to be localized at the inner core of the MB calyces in the brains of larvae and early pupae and then differentiate into sKCs in the brains of late pupae^55,56^, we assume that these pupa-specific p-Mblk-1immunoreactive cells were the MB neuroblasts. No significant signals, however, were detected at inner core inside the MB calyces in some pupal brains (Fig. 7A–D), possibly because these pupae were older than others (Fig. 5D), and thus almost all MB neuroblasts had already differentiated into sKCs and there were only few neuroblasts in the MBs of these pupae. In the MBs, Mblk-1-like immunoreactivity was detected in both the nuclei and cytoplasm of the somata of KCs in the brains of nurse bees, foragers, and re-orienting bees (Figs. 6 and S7). Similarly, p-Mblk-1-like immunoreactivity was also detected in both the nuclei and cytoplasm of the somata of KCs in the brains of late pupal workers (Fig. 7), suggesting that Mblk-1 functions in transcription in both pupal and adult stages and that p-Mblk-1 does not necessarily represent an active form of a transcription factor, unlike Ikaros^49^ and SOX11^50^.

Taken together, our findings suggest that Mblk-1 functions in the lKCs in both pupal and adult stages and that p-Mblk-1 has pupal stage-specific functions in the MB neuroblasts and lKCs in the honey bee brain. It is possible that both Mblk-1 and p-Mblk-1 function in morphogenesis during pupal stages, whereas Mblk-1 changes its function to have specific roles, including that in learning and memory, in the lKCs in the adult brain. Further studies are needed to identify the target genes of Mblk-1 and p-Mblk-1 in both pupal and adult brains using chromatin immunoprecipitation-sequence analysis with the anti-fragment A and anti-p-Mblk-1 antibodies.

## Supplementary information

Supplementary information

## Data Availability

All data generated or analyzed during this study are included in this published article (and its Supplementary Information files).
