# Publisher Correction: Developmental stage-specific distribution and phosphorylation of Mblk-1, a transcription factor involved in ecdysteroid-signaling in the honey bee brain

**DOI:** 10.1038/s41598-020-68540-y

**Published:** 2020-07-09

**Authors:** Hitomi Kumagai, Takekazu Kunieda, Korefumi Nakamura, Yasuhiro Matsumura, Manami Namiki, Hiroki Kohno, Takeo Kubo

**Affiliations:** Department of Biological Sciences, Graduate School of Science, The University of Tokyo, Bunkyo-ku, Tokyo 113-0033 Japan

Correction to: *Scientific Reports* 10.1038/s41598-020-65327-z, published online 26 May 2020

The version of this Article previously published contained a corrupted version of Figure 5. This has now been corrected in the PDF and HTML versions of the paper.

