# Supplementary material for: Developmental stage-specific distribution and phosphorylation of Mblk-1, a transcription factor involved in ecdysteroid-signaling in the honey bee brain
Source: Sci Rep. 2020 May 26;10:8735. doi: 10.1038/s41598-020-65327-z (PMC7250831; doi:10.1038/s41598-020-65327-z)
Supplement: Supplementary file 1 — Supplementary information [file 41598_2020_65327_MOESM1_ESM.pdf]

Developmental stage-specific distribution and phosphorylation of Mblk-1, a transcription factor involved in ecdysteroid-signaling in the honey bee brain

Hitomi Kumagai[1]†, Takekazu Kunieda[1]†, Korefumi Nakamura[1]†, Yasuhiro Matsumura[1]†, Manami Namiki[1], Hiroki Kohno[1] and Takeo Kubo[1]*

[1]: Department of Biological Sciences, Graduate School of Science, The University of Tokyo, Bunkyo-ku, Tokyo 113-0033, JAPAN

†: Equal contribution.

*: Corresponding author, stkubo@bs.s.u-tokyo.ac.jp

Running title: Expression and phosphorylation of Mblk-1.

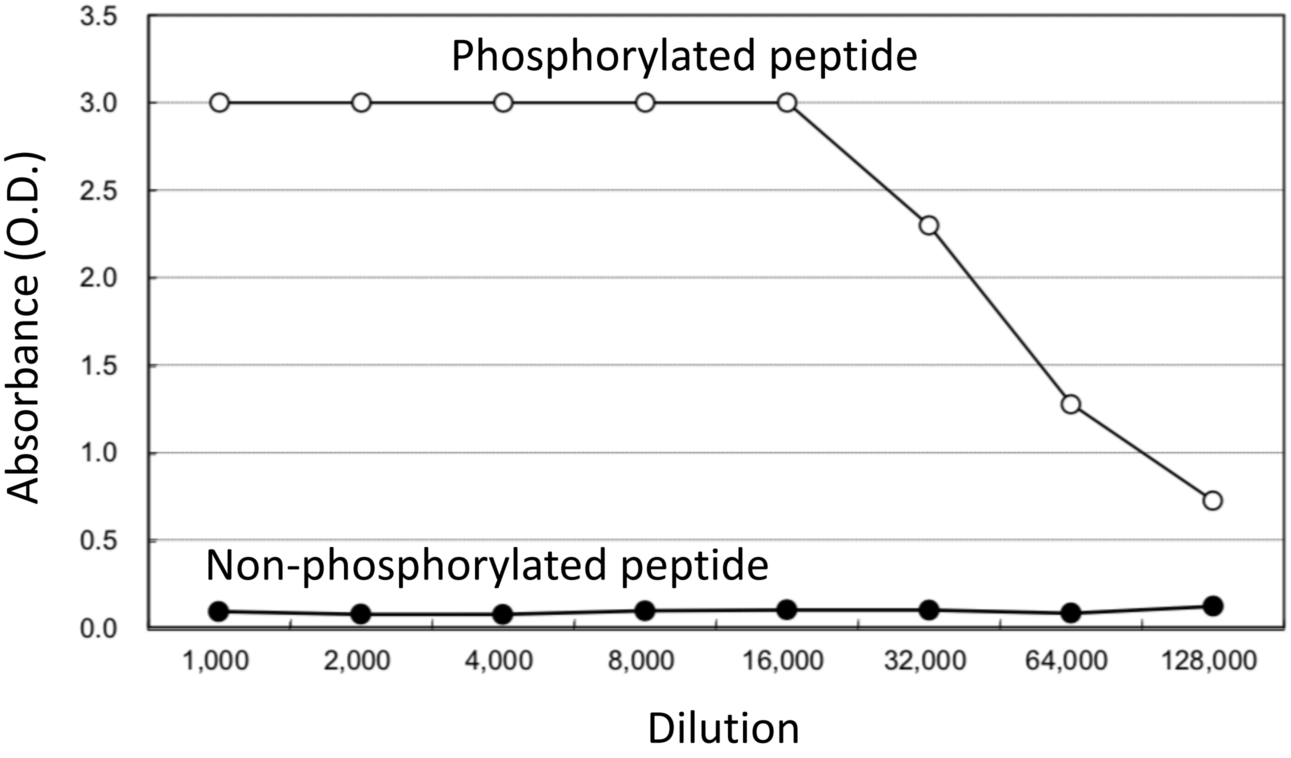

Phosphorylated peptide
Non-phosphorylated peptide
Absorbance (O.D.)
3.5
3.0
2.5
2.0
1.5
1.0
0.5
0.0
1,000
2,000
4,000
8,000
16,000
32,000
64,000
128,000
Dilution

Figure S1. Specific reactivity of the purified anti-p-Mblk-1 antibody against the phosphorylated synthetic peptide. Reactivity of the affinity-purified anti-p-Mblk-1 antibody was examined by enzyme-linked immunosorbent assay. As antigens, the phosphorylated peptide or the non-phosphorylated peptide were immobilized in a microtiter plate and were reacted with serial dilutions of the antibody. The amount of the captured antibody was quantified by the absorbance of the product generated by the enzyme linked to the secondary antibody. The antibody exhibited a substantial reactivity against the phosphorylated peptide, whereas almost no reactivity was detected against the non-phosphorylated peptide.

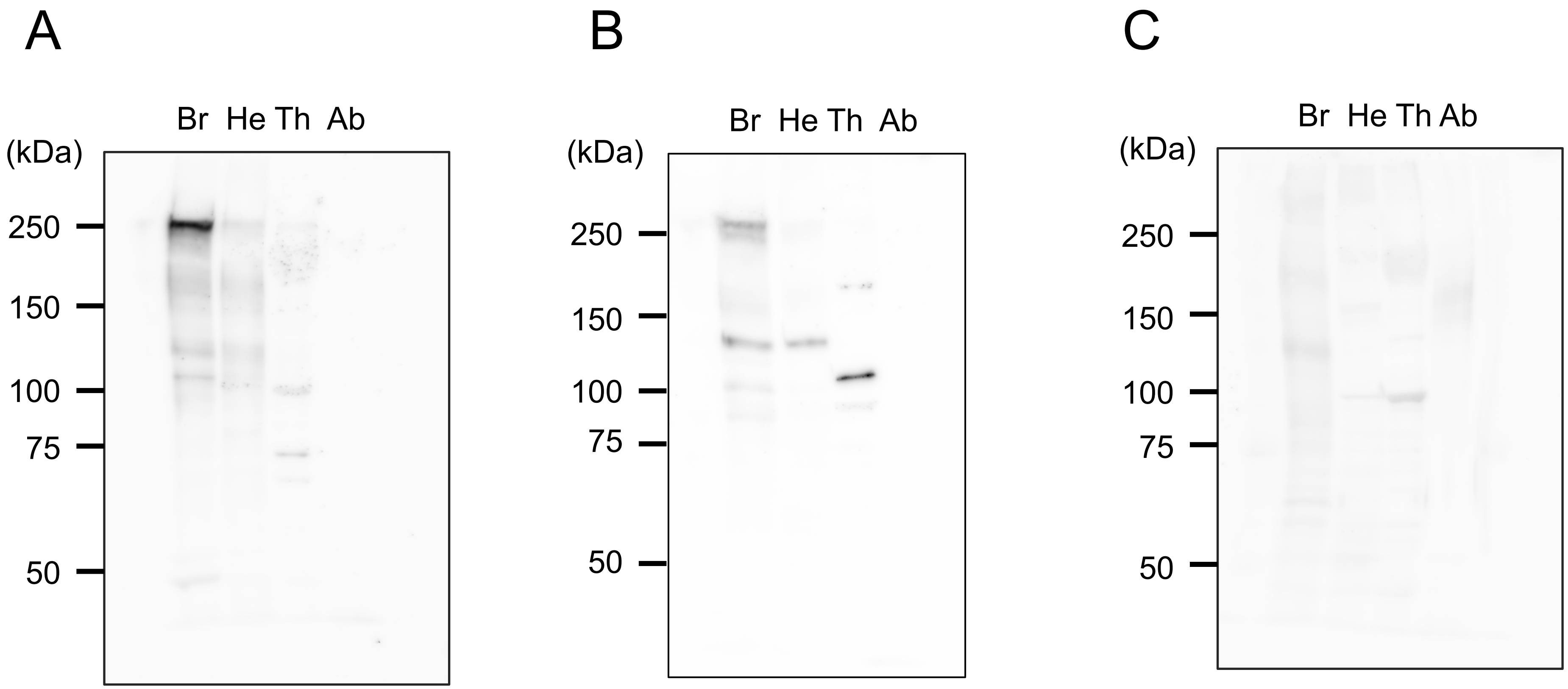
A
Br He Th Ab
(kDa)
250
150
100
75
50
B
Br He Th Ab
(kDa)
250
150
100
75
50
C
Br He Th Ab
(kDa)
250
150
100
75
50

Figure S2. The uncropped data which correspond to Figure 1 panels (B) to (D). The raw data, containing the entire signals and obtained with exposure time 30 sec for Figure 1 panels (B) to (D), are presented.

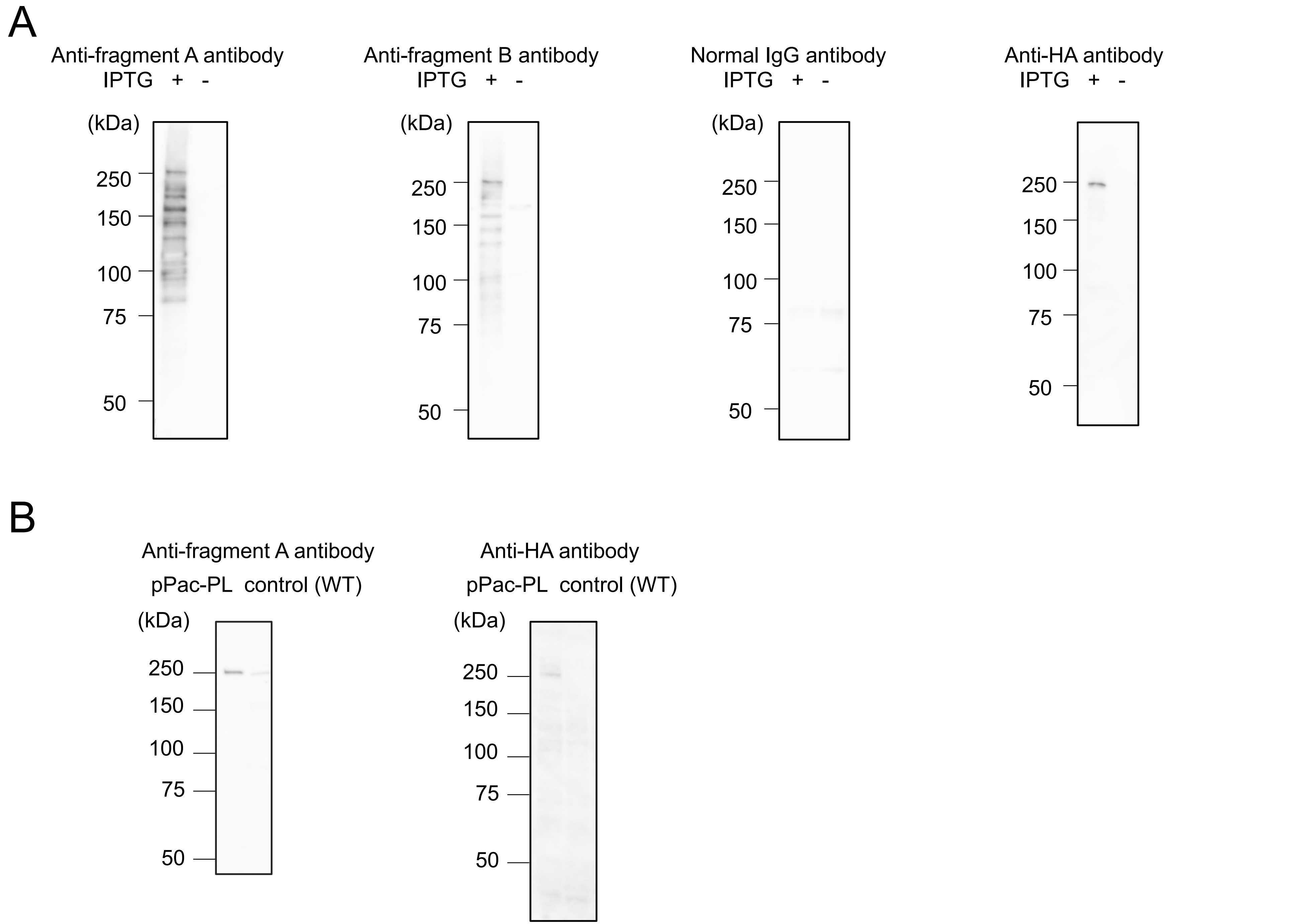
A
Anti-fragment A antibody
IPTG + -
(kDa)
250
150
100
75
50
Anti-fragment B antibody
IPTG + -
(kDa)
250
150
100
75
50
Normal IgG antibody
IPTG + -
(kDa)
250
150
100
75
50
Anti-HA antibody
IPTG + -
250
150
100
75
50
B
Anti-fragment A antibody
pPac-PL control (WT)
(kDa)
250
150
100
75
50
Anti-HA antibody
pPac-PL control (WT)
(kDa)
250
150
100
75
50

Figure S3. The uncropped data which correspond to Figure 2 panels (A) and (B). (A) The raw datum, containing the entire signals and obtained with exposure time 20 sec, 50 sec, 50 sec, and 100 sec, respectively from the left side, for Figure 2 panels (A) is presented. (B) The raw datum, containing the entire signals and obtained with exposure time 150 sec and 580 sec, respectively from the left side, for Figure 2 panels (B) is presented.

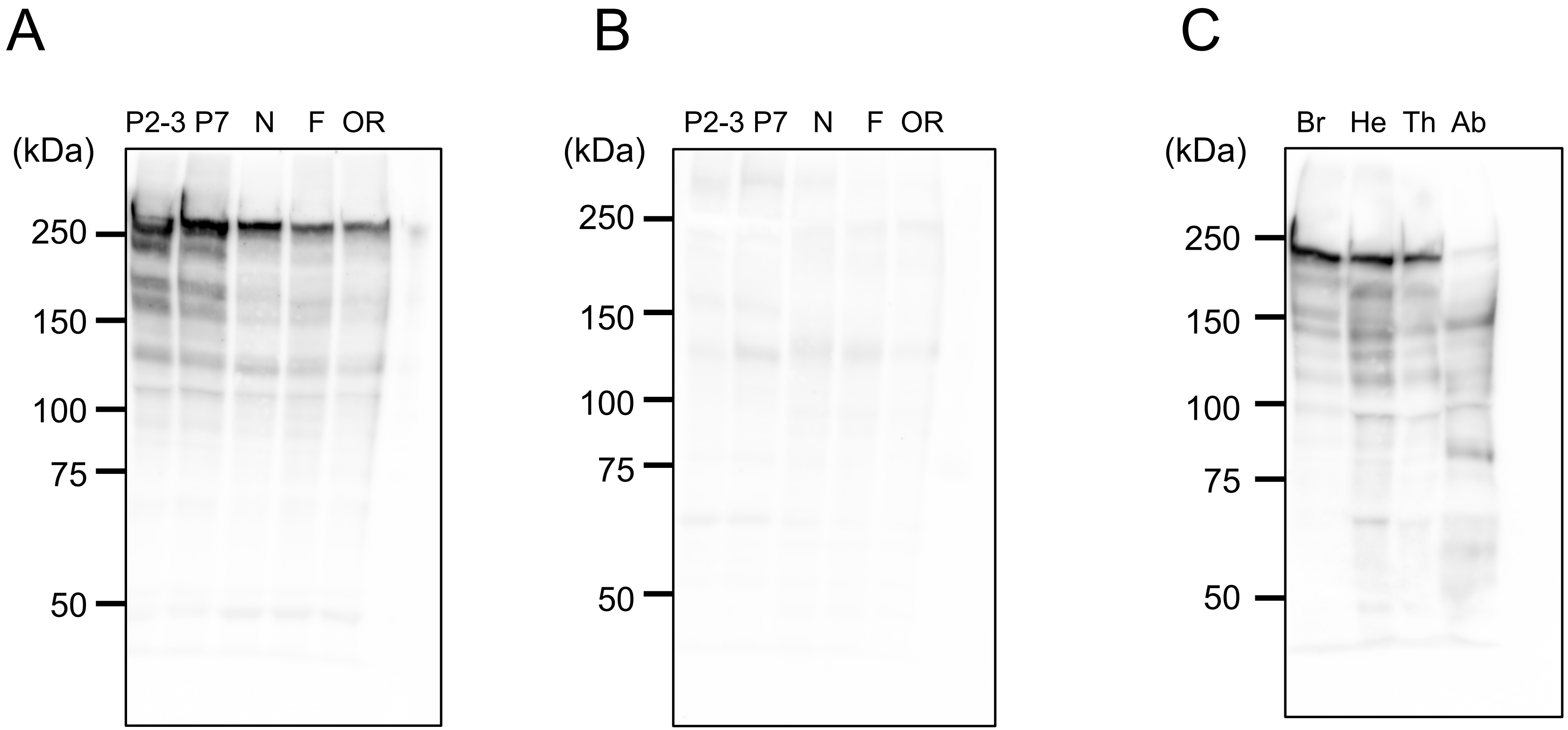
A
P2-3 P7 N F OR
(kDa)
250
150
100
75
50
B
P2-3 P7 N F OR
(kDa)
250
150
100
75
50
C
Br He Th Ab
(kDa)
250
150
100
75
50

Figure S4. The uncropped data which correspond to Figure 3 panels (A) to (C). The raw data, containing the entire signals and obtained with exposure time 30 sec for Figure 3 panels (A) to (C), are presented.

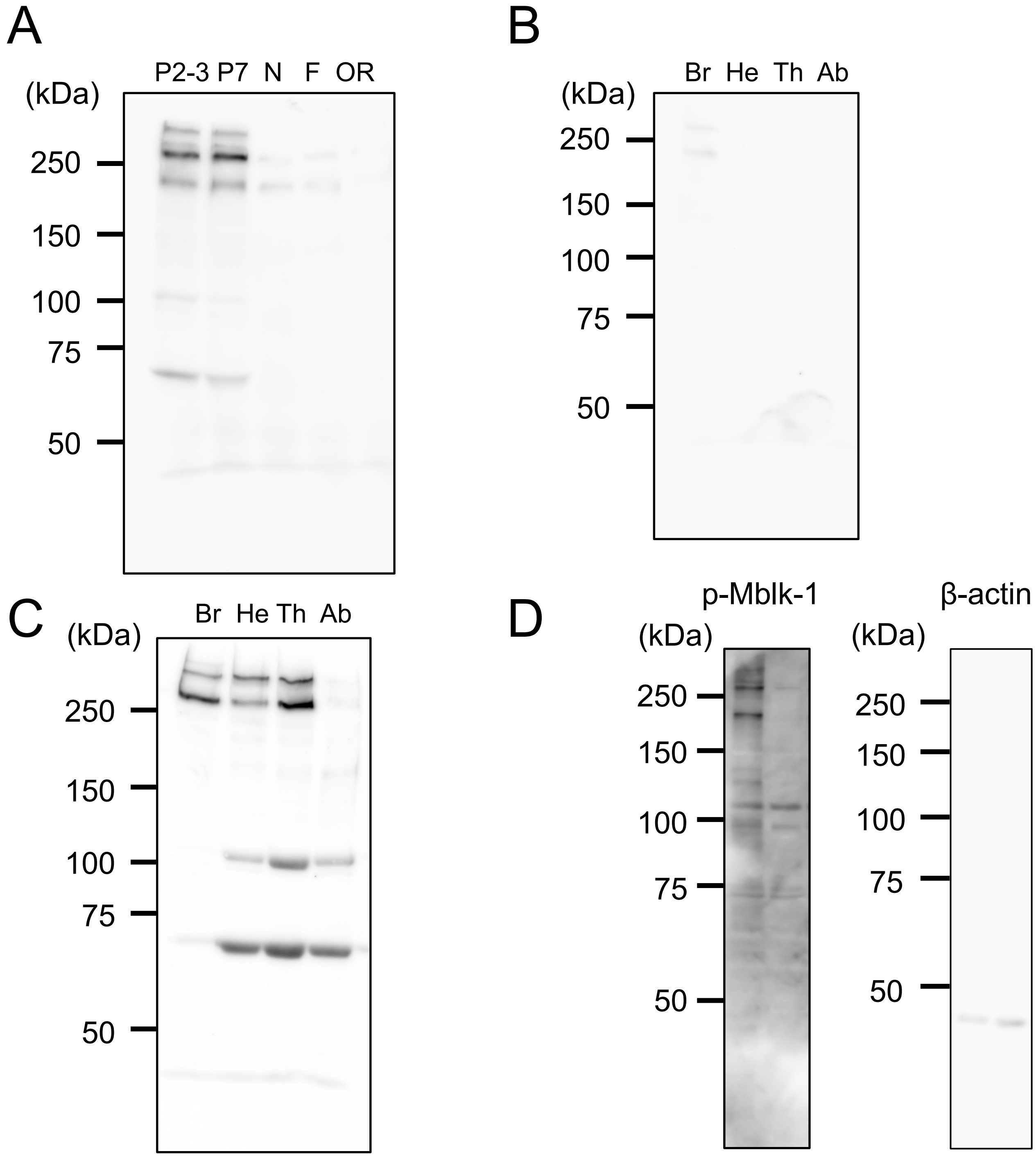
A
P2-3 P7 N F OR
(kDa)
250
150
100
75
50
B
Br He Th Ab
(kDa)
250
150
100
75
50
C
Br He Th Ab
(kDa)
250
150
100
75
50
D
p-Mblk-1
β-actin
(kDa)
250
150
100
75
50
(kDa)
250
150
100
75
50

Figure S5. The uncropped data which correspond to Figure 4 panels (A) to (D). The raw data, containing the entire signals and obtained with exposure time 30 sec for Figure 4 panels (A) to (C), 40 sec and 60 sec for (D, p-Mblk-1 and D, β actin), are presented.

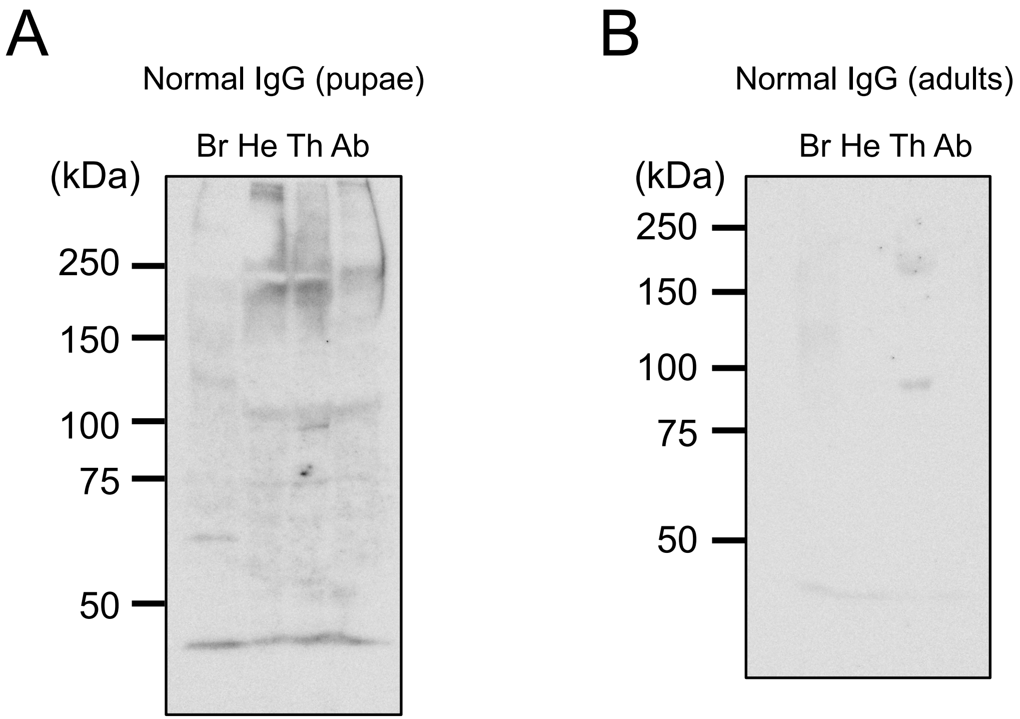
A
Normal IgG (pupae)
Br He Th Ab
(kDa)
250
150
100
75
50
B
Normal IgG (adults)
Br He Th Ab
(kDa)
250
150
100
75
50

Figure S6. Immunoblotting analysis in various body parts using normal IgG as a negative control. (A) A control experiment using normal IgG for immunoblotting analysis of p-Mblk-1 in the lysate of brains (Br), heads without brains (He), thoraxes (Th), and abdomens (Ab) of worker pupae. (B) A control experiment using normal IgG for immunoblotting analysis of p-Mblk-1 in the lysate of brains (Br), heads without brains (He), thoraxes (Th), and abdomens (Ab) of adult workers. Note that, the raw data, containing the entire signals, which were obtained by re-probing with normal IgG and the same membrane used in Figure 4 panels (B) and (C) are uncropped and grouped as Figure S6. Although there was some non-specific staining in panels (A) and (B), none of them corresponded to signals detected with anti-p-Mblk-1 antibody shown in Figure 4. The exposure times for both panels (A) and (B) were 30 sec.

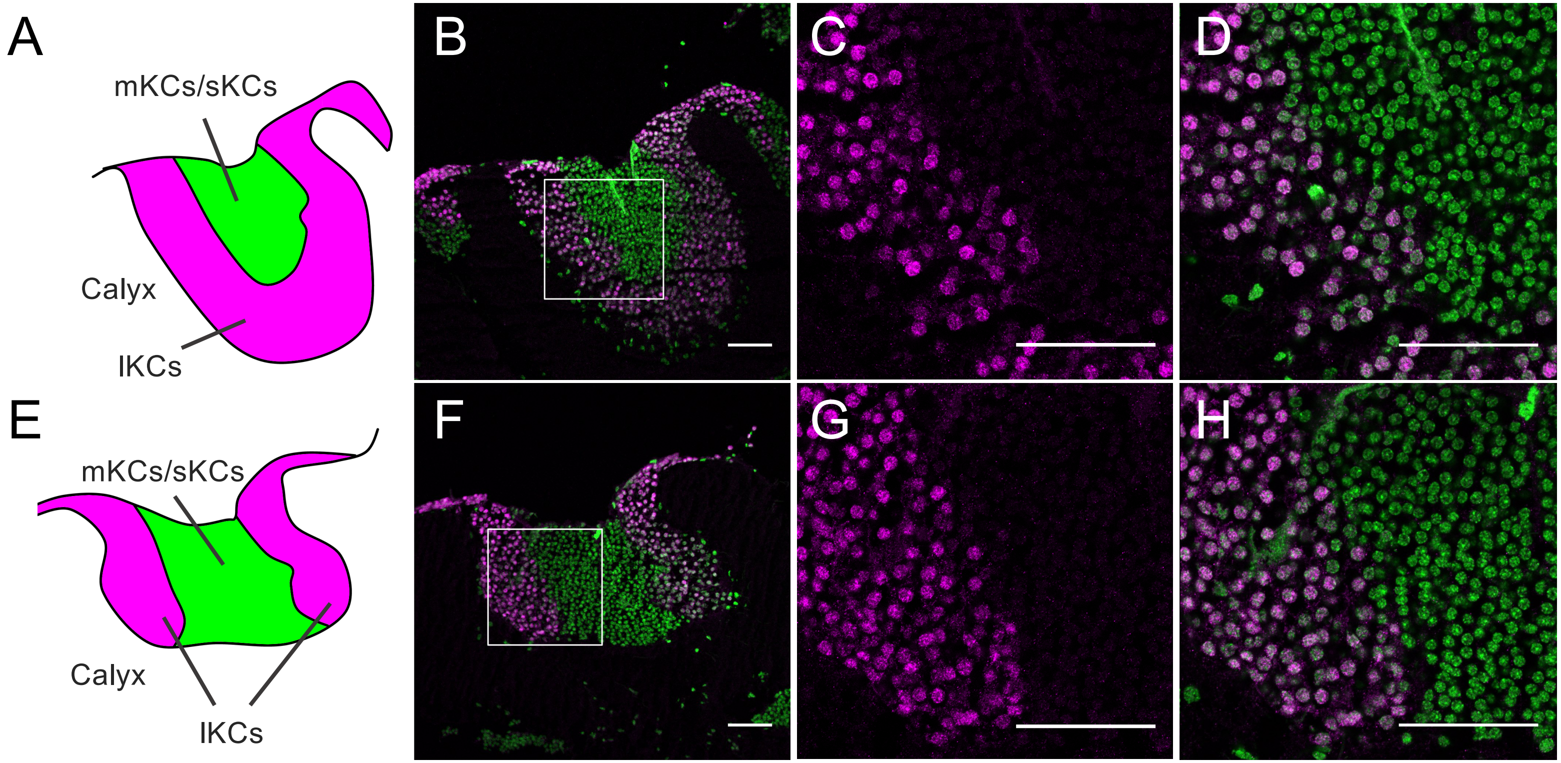
A
mKCs/sKCs
Calyx
lKCs
B
C
D
E
mKCs/sKCs
Calyx
lKCs
F
G
H

Figure S7. Immunohistochemistry of Mblk-1 in the brains of a nurse bee and a forager. (A-H) Immunohistochemistry of Mblk-1 in the brains of a nurse (A-D) and a forager (E-H). (A and E) Schematic drawings of the MB calyces shown in panels (B) and (F), respectively. lKCs, large-type KCs; mKCs, middle-type KCs; sKCs, small-type KCs. (B and F) Immunohistochemistry of Mblk-1 in the brains of a nurse bee (B) and a forager (F). (C-D and G-H) Magnified views of boxed area shown in panels (B) and (F), respectively. Signals for fluorescence of Mblk-1 and DAPI are shown in magenta and green, respectively. Bars indicate 50 μm.

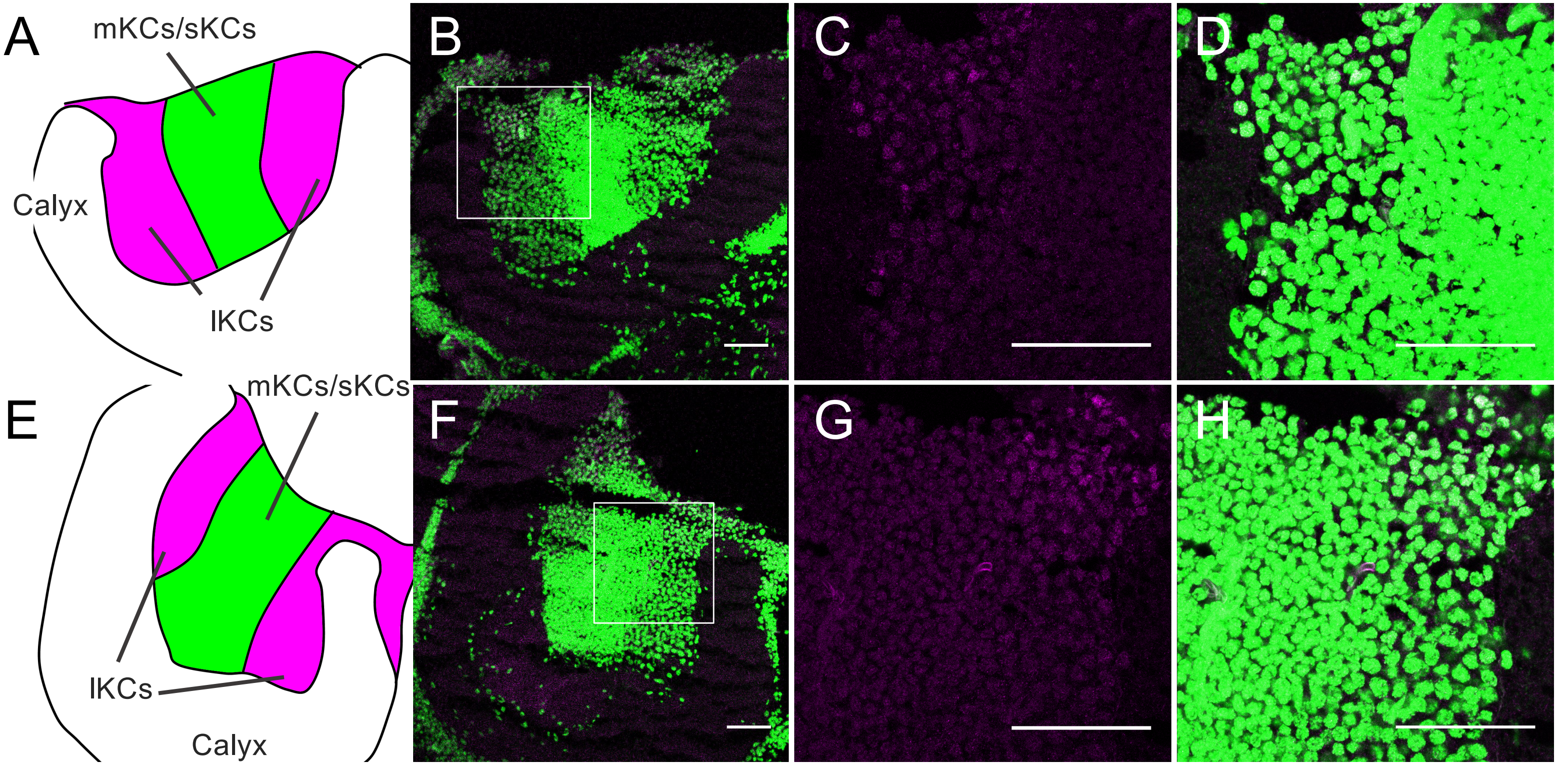
A
mKCs/sKCs
Calyx
lKCs
B
C
D
E
mKCs/sKCs
lKCs
Calyx
F
G
H

Figure S8. Immunohistochemistry of p-Mblk-1 in the brains of a nurse bee and a forager. (A-H) Immunohistochemistry of p-Mblk-1 in the brains of a nurse bee (A-D) and a forager (E-H). (A and E) Schematic drawings of the MB calyces shown in panels (B) and (F), respectively. lKCs, large-type KCs; mKCs, middle-type KCs; sKCs, small-type KCs. (B and F) Immunohistochemistry of p-Mblk-1 in the brains of a nurse bee (B) and a forager (F). (C-D and G-H) Magnified views of boxed area shown in panels (B) and (F), respectively. Signals for fluorescence of p-Mblk-1 and DAPI are shown in magenta and green, respectively. Bars indicate 50 μm.
